# Mild Phenotypes of Gyrate Atrophy in a Heterozygous Carrier with One Variant Allele of *OAT*

**DOI:** 10.3390/genes15081020

**Published:** 2024-08-02

**Authors:** Yuqiao Ju, Yuan Zong, Xiao Li, Fengjuan Gao, Qing Chang, Xin Huang

**Affiliations:** 1 Eye Institute and Department of Ophthalmology, Eye and ENT Hospital of Fudan University, Shanghai 200031, China; 2 NHC Key Laboratory of Myopia and Related Eye Diseases, Key Laboratory of Myopia and Related Eye Diseases, Chinese Academy of Medical Sciences, Shanghai 200031, China; 3 Shanghai Key Laboratory of Visual Impairment and Restoration, Shanghai 200031, China

**Keywords:** gyrate atrophy of the choroid and retina (GACR), *OAT* gene, carrier, allele, autosomal recessive

## Abstract

This study aimed to identify whether gyrate atrophy of the choroid and retina (GACR) heterozygous individuals have possible clinical manifestations and to explore the potential pathogenic mechanism. In this retrospective study, we surveyed a two-generation pedigree of an individual diagnosed with GACR. Two family members underwent ophthalmological, hematologic, and genetic tests. An arginine-restricted diet with vitamin B6 supplementation was implemented; clinical assessments were repeated every 3 months during follow-up. The relative *OAT* mRNA expression was determined using a real-time quantitative polymerase chain reaction. The 19-year-old compound heterozygous daughter (*OAT*: c.1186C>T; c.748C>T) had bilateral pathologic myopia, posterior staphyloma, chorioretinal atrophy, macular abnormalities, and elevated hematologic ornithine. The 54-year-old heterozygous mother (*OAT*: c.1186C>T) presented with bilateral pathologic myopia, asymmetric posterior staphyloma, retina and choroidal capillary layer atrophy, retinal pigment epithelium abnormalities, and mildly elevated hematologic ornithine. Compared to normal individuals, the daughter and mother had 29% and 46% relative *OAT* mRNA expression, respectively (*p* < 0.001). We believe that this is the first report of a carrier of one *OAT* variant allele exhibiting a mild phenotype, suggesting that family members should be aware of the possibility of clinical involvement in carriers with some autosomal recessive conditions. Additional data suggest that nonsense-mediated, decay-initiated mRNA degradation may cause GACR.

## 1. Introduction

Gyrate atrophy of the choroid and retina (GACR; OMIM#258870) is a rare autosomal recessive metabolic disease [1], with an estimated worldwide prevalence of approximately 1 per 2,770,000 to 1 per 1,500,000, except in the Finnish population [2]. Since it was first described by Fuchs in 1896, only three patients from two families have been reported in China [3].

The *OAT* (OMIM#613349) gene on chromosome 10q26.13, which encodes ornithine-δ-aminotransferase, is responsible for the conversion of ornithine (Orn) to glutamate and is the causative gene of GACR. Variants in the *OAT* gene can result in OAT deficiency and the excessive accumulation of Orn [4]. The elevated serum Orn levels are toxic to the retina and choroid, causing chorioretinal degeneration and atrophy [5]. As a severe vision-threatening disease, nyctalopia and decreased visual acuity early in childhood are common symptoms in patients with GACR. Eventually, the disease leads to severe visual impairment and functional blindness at 40–50 years of age. Reduced electroretinogram responses and macular abnormalities are also frequently observed in GACR [6,7,8,9]. In addition, extraocular manifestations in the muscle and nervous systems have also been reported, but not consistently. Clinically, typical fundus manifestations of multiple, sharply demarcated, scallop-shaped chorioretinal atrophy; an elevated serum Orn level; and genetic testing can help diagnose GACR. Currently, a protein-restricted diet focusing on lowering plasma Orn levels or supplementation with pyridoxine (vitamin B6) is experimentally administered to patients with GACR to prevent vision loss [10,11,12].

In autosomal recessive diseases, individuals with a heterozygous variant can maintain normal biological conditions and have a normal phenotype according to Mendel’s Law of Dominance [13]. However, in some autosomal recessive genetic metabolic disorders, including phenylketonuria, cystic fibrosis, and Usher syndrome, a few carriers can display mild symptoms [14,15,16,17]. Although this is uncommon, it indicates that appropriate clinical examinations should be suggested for this population group.

To date, no reports have identified carriers with GACR symptoms. Here, we report a GACR family within two generations: a daughter with compound heterozygous variants and mother with a heterozygous variant in *OAT*, both of whom exhibited manifestations of GACR, while the mother presented with a milder phenotype. The potential pathogenic mechanisms of *OAT* nonsense variants and the relationship between the variants and clinical manifestations were analysed to improve understanding of GACR pathogenesis.

## 2. Materials and Methods

### 2.1. Patients

The proband from a two-generation pedigree diagnosed with GACR at our clinic in August 2023 was enrolled in this retrospective study. Approval for data collection and analysis was granted by the Institutional Review Board of the Eye and ENT Hospital of Fudan University, Shanghai, China. Written informed consent, consistent with the Declaration of Helsinki, was acquired from the participants before performing all examinations and genetic testing.

### 2.2. Clinical Examinations, Biochemical Analysis, and Treatments

Two participants from one family underwent standard ophthalmological examinations, including slit-lamp biomicroscopy (Keeler, Windsor, UK), best-corrected visual acuity (BCVA, Snellen Chart), intraocular pressure (NCT, Canon TX-20, Saitama Prefecture, Japan), ultra-widefield fundus and fundus autofluorescence imaging (Optos UWF™, Scotland, UK), spectral domain optical coherence tomography (OCT; SD-OCT, Heidelberg Engineering, Heidelberg, Germany), optical coherence tomography angiography (VG200S, Svision Imaging, Ltd., Luoyang, China), and full-field electroretinography (RetiMINERTM, IRC, Chongqing, China). Axial length was measured using IOL Master (IOLMaster 700, Carl ZEISS, Oberkochen, Germany). Refraction was assessed by both autorefraction and subjective refraction, and the spherical equivalence was calculated as spherical dioptre (D) + ½ cylindrical dioptric power. High myopia was defined as axial length (AL) ≥ 26.00 mm or ≤−6.0 D spherical equivalent (SE). Posterior staphyloma was classified according to Curtin’s and Kyoko’s systems [18,19]: the contour of the outermost border of the posterior staphyloma was analysed using ultra-widefield fundus, fundus autofluorescence, and B-ultrasound. Of these, Type I corresponds to wide macular staphyloma, and Type V refers to inferior staphyloma. Pathologic myopia is defined by a presence of typical complications in the fundus, including posterior staphyloma and myopic maculopathy, occurring with or without high myopia. [20]. Peripheral blood metabolic tests were performed using a GCMS-QP2010 (Shimadzu, Kyoto, Japan) and an ACQUITY TQ-D instrument (Waters Corp., Milford, MA, USA). Therapy with an arginine-restricted diet and vitamin B6 supplementation was conducted as previously described, and clinical examinations were repeated every 3 months during the follow-up from August 2023 [10,11,12].

### 2.3. Genetic Testing and Pathogenicity Assessment

For whole Exome sequencing (WES), peripheral blood samples from both participants were collected in EDTA-anticoagulant tubes and then outsourced to Shanghai WeHealth Biomedical Technology Co., Ltd., Shanghai, China. The genomic DNA was subjected to exome sequencing, comprising more than 23,000 genes, as previously reported [21]. All reads were aligned to the National Centre for Biotechnology Information (https://www.ncbi.nlm.nih.gov/ accessed on 10 August 2023) human reference genome GRCh38/hg19. The amplified genomic sequences were compared with the *OAT* reference sequence NM_000274.4.

For whole genome sequencing (WGS), the genome sequencing was conducted by Shanghai WeHealth Biomedical Technology Co., Ltd., as previously reported [22]. The TWIST Library Preparation EF Kit 2.0 (104207) was used for DNA fragmentation and genome library construction.

For Sanger sequencing, the detected variants were further validated by Sanger sequencing as previously reported [21]. Polymerase chain reaction (PCR) primers were designed using Primer3Plus (http://primer3plus.com/cgi-bin/dev/primer3plus.cgi accessed on 10 August 2023).

### 2.4. In Silico Analysis and Protein Structure Modelling

We reviewed publications in the 1000 Genome database, ExAC, gnomAD, PubMed (https://pubmed.ncbi.nlm.nih.gov/ accessed on 20 August 2023), and the Exome Sequencing Project (http://evs.gs.washington.edu/EVS/ accessed on 20 August 2023) to search for population frequencies and determine if the variants had been previously reported. The pathogenicity of the identified *OAT* variants was predicted using various in silico prediction tools, including LRT, FATHMM-MKL, ClinPred, CADD, DANN, GERP, Mutation Taster, and SPIDEX, as previously described [21]. All variants were subsequently evaluated according to the American College of Medical Genetics and Genomics (ACMG) guidelines [23].

Three-dimensional structural modelling of the OAT protein was performed using PyMOL (https://pymol.org/2/ accessed on 1 October 2023), based on a known structure (PDB ID: 7T9Z). To analyse variant conservation, the amino acid sequence of OAT in different species was obtained from UniProt (https://www.uniprot.org/ accessed on 1 October 2023), and a multiple-sequence alignment analysis of the variant sites in several species was performed using DNAMAN 6.0 software (https://www.lynnon.com/download/ accessed on 1 October 2023).

### 2.5. OAT mRNA Analysis

Whether nonsense variants impact gene transcription was confirmed by detecting the relative gene expression levels of *OAT* cDNA utilizing real-time fluorescence quantitative PCR (RT-qPCR).

The total RNA was extracted from blood samples of the patients and three healthy controls using an RNA extraction kit (TIANGEN, Beijing, China). RNA from whole blood samples was reverse-transcribed to cDNA using the Hifair^®^-II-1st-Strand cDNA-Synthesis Kit (Yeasen, Beijing, China). Normal control samples were obtained from healthy volunteers without pathogenic *OAT* variants. RNA was then reverse-transcribed into cDNA. The targeted RNA was amplified by qPCR using the SYBR Green PCR master mix (Novoprotein, Shanghai, China) and then analysed on an RT-qPCR System (Roche, Shanghai, China). The primers used are listed in Table S1. The relative *OAT* mRNA expression was calculated and normalized against an internal reference gene *GAPDH* based on the 2^−ΔΔCt^ method [24]. All experiments were performed in triplicate. The *OAT* cDNA of two family members and the normal controls were further validated by Sanger sequencing and Next-generation Sequence (NGS), as previously reported [21]. The amplified genomic sequences were compared with the *OAT* reference sequence NM_000274.4. Polymerase chain reaction (PCR) primers were designed using Primer3Plus 6.0 (http://primer3plus.com/cgi-bin/dev/primer3plus.cgi accessed on 1 May 2024).

### 2.6. Statistical Analyses

The statistical analysis of the relative *OAT* mRNA expression was performed using one-way ANOVA, followed by Bonferroni’s multiple-comparison correction, using GraphPad Prism 9 (Boston, MA, USA) (https://www.graphpad.com/ accessed on 11 October 2023). *p* < 0.05 was considered statistically significant.

## 3. Results

### 3.1. Ocular Characteristics

**Figure 1:**
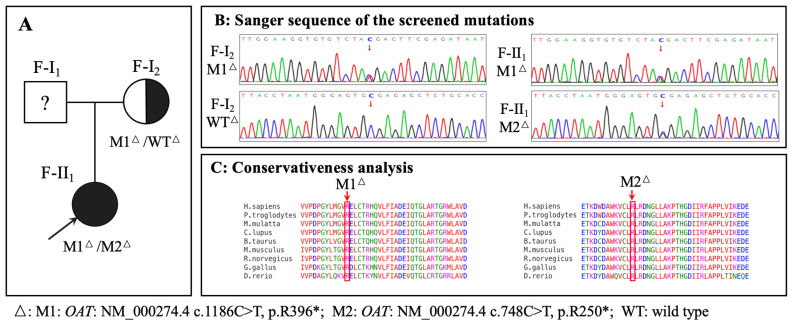
Identification of *OAT* variants in the family. (**A**): Pedigree of the family with gyrate atrophy with choroid and retina (GACR). Question marks indicate that the data were unavailable. (**B**): Sanger sequencing of the identified variants. Variant or wild-type nucleotides are indicated by red arrows. (**C**): Conservative analysis.

**Figure 2:**
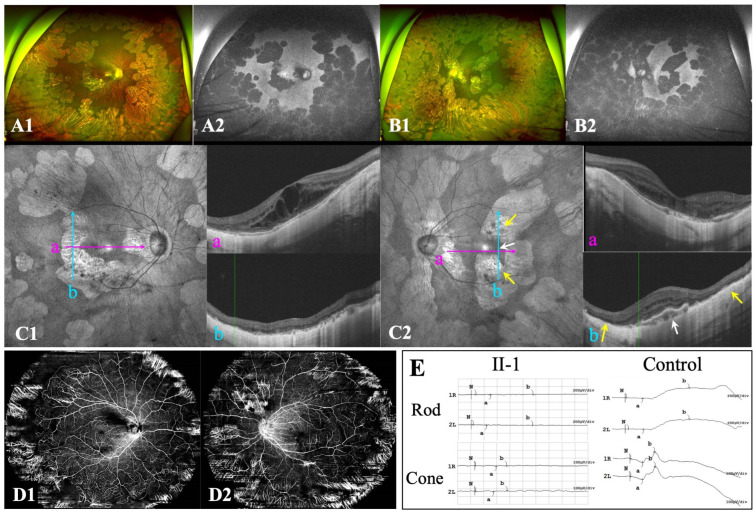
Ocular characteristics of F-II_1_. (**A**,**B**): Ultra-widefield fundus images, including colour fundus and autofluorescence imaging. (**C**): Optical coherence tomography (OCT) revealed macular abnormalities (**C1a**,**C2a**) as well as retinal and choriocapillaris atrophy (**C1b**,**C2b**) in both eyes. An OCT B-scan of the left eye revealed deposits below the retinal pigment epithelium cells and choroidal atrophy (**C2b**). (**D**): Wide angio-OCT (OCTA) images of F-II_1_ in the right (**D1**) and left eye (**D2**). (**E**): Electroretinography of F-II_1_ cells and normal controls. Yellow arrows indicate retinal and choriocapillaris atrophy. White arrows indicate deposits below the retinal pigment epithelium cells.

The proband (F-II_1_) (Figure 1A), a 19-year-old young lady, visited our clinic complaining of significant bilateral vision loss. In addition to a history of high myopia and regular visits to optometry doctors since childhood, the patient had no known systemic or ocular disorders. BCVA was 20/66 in both eyes, with a spherical equivalence refraction error of −17.00 D and −18.25 D in the right and left eye, respectively. Ophthalmological examination revealed punctate posterior subcapsular cataracts in the right eye (Figure S1). Both eyes presented with wide, macular posterior staphyloma and were classified as Type 1 bilaterally. Multiple, sharply demarcated, scallop-shaped chorioretinal atrophy was found in the mid-peripheral and peripheral regions of the fundus in both eyes (Figure 2A,B). Enhanced-depth OCT showed increased central macular thickness with multiple intraretinal cystic spaces and bilateral chorioretinal atrophy (Figure 2(C1,C2)). The extensive loss of the outer layers and significant thinning of the retina are indicated by yellow arrows (Figure 2(C2)). OCT imaging of the left eye revealed hyperreflective deposits below the retinal pigment epithelium (RPE) (Figure 2(C2b), white arrows), whereas the corresponding region on the fundus image appeared relatively normal. Wide optical coherence tomography angiography images showed significant bilateral vascular loss (Figure 2(D1,D2)). An electroretinography examination revealed a nearly extinguished bilateral rod and cone response (Figure 2E).

**Figure 3:**
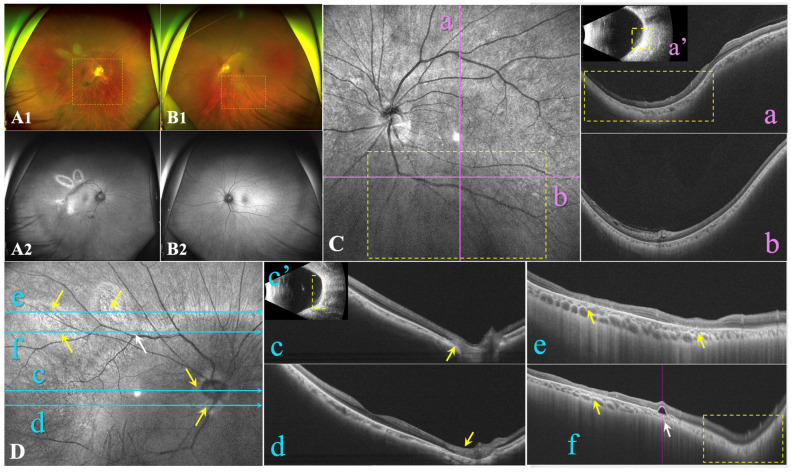
Ocular characteristics of F-I_2_. (**A**,**B**): Ultra-widefield fundus images include both colour fundus and autofluorescent images. (**C**): Optical coherence tomography (OCT) B-scan (**Ca**,**b**) and B-ultrasound (**Ca’**) of the left eye revealed inferior posterior staphyloma (PS) with retinoschisis. (**D**): OCT B-scan (**Dc**,**f**) and B-ultrasound (**Dc’**) of the right eye revealed a wide macular PS. OCT B-scan of the right eye revealed focal circular areas of retinal atrophy and choriocapillaris atrophy located in the region adjacent to the disc (**Dc**,**d**) and the supertemporal vascular arcade (**De**,**f**), with pigment epithelial detachment (PED) close to the retinal atrophy lesion (**Df**). Yellow arrows refer to focal circular areas of retinal atrophy and choriocapillaris atrophy. Yellow dotted squares indicate the site of PS. White arrows indicate PED.

As the mother (F-I_2_) (Figure 1A) presented with unusual unilateral high myopia and fluttering dark shadows in her right eye, an ophthalmological examination was also conducted. She had no history of systemic or ocular disorders other than myopia. She also denied any family history of ocular or visual problems, including myopia or severe visual impairment. BCVA was 20/28 in both eyes with a spherical equivalence refraction error of −9.00 D and −2.75 D in the right and left eye, respectively. Her right eye presented with a wide, macular posterior staphyloma (Type 1) (Figure 3(A1,Dc’), yellow dotted square), and the axial length was 26.45 mm. Furthermore, focal circular areas of retinal atrophy were super-temporally observed in her right eye, and fundus autofluorescence imaging revealed that the corresponding area was hypoautofluorescent surrounded by hyperautofluorescent areas (Figure 3A). An OCT B-scan demonstrated atrophy of the retina and choroidal capillary layers in the parapapillary and supratemporal retina (Figure 3(Dc–f)), yellow arrow). Pigment epithelial detachment (PED) and an area of disorganized RPE layer were detected at the edge of one cilioretinal atrophy lesion, close to the superior vascular arch (Figure 3(Df), white arrow). However, the left eye also presented with pathologic myopia and an axial length of 24.52-mm. An inferior posterior staphyloma (Type 5) (Figure 3(B1,Ca,b), yellow dotted square) with regional retinoschisis (Figure 3(Cb)) was found on fundus imaging, B-scan, and OCT examination. More detailed ocular examination results are displayed in Table 1. Unfortunately, the daughter’s father was not available for ocular examination or genetic testing.

### 3.2. Hematologic Biochemical Findings

Based on the clinical findings, serum amino acid levels were tested for both members (Table 1). Consistent with our expectations, the daughter’s (F-II_1_) Orn level showed a marked elevation (257.92 µM, reference range: 10–100 µM) and the creatine level was slightly decreased to 94.83 µM (reference range: 95–1000 µM). Surprisingly, the mother’s (F-I_2_) Orn level was slightly above the population reference range as 102.08 µM.

### 3.3. Genetic Findings

**Figure 4:**
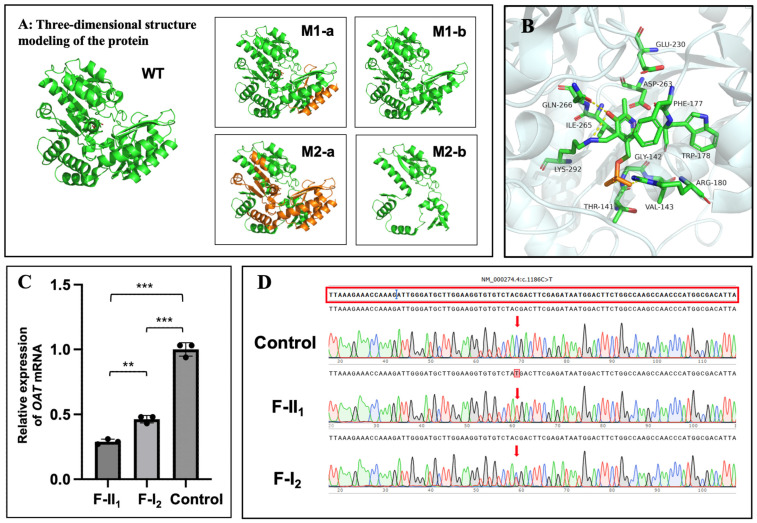
Protein structure modelling and transcription analysis. (**A**): Three-dimensional structural model of the OAT protein. The truncated portion of the protein is coloured orange, and the mutants are shown as M1-b and M2-b. WT: wild-type. (**B**): Catalytic sites for substrate specificity in the OAT protein. Capital letters correspond to the abbreviations of different amino acid names, and numbers correspond to amino acid orders. (**C**): Relative *OAT* mRNA expression levels in peripheral blood (the whole blood samples) of the two family members and three normal controls. **: *p* < 0.01; *** *p* < 0.001. *p* < 0.05 was considered statistically significant. (**D**): Sanger sequencing of *OAT* cDNA reverse-transcribed from the mRNA of the whole blood samples on M1 (c.1186C>T) in the normal controls and two family members.

Exome sequencing and Sanger sequencing identified a compound heterozygous *OAT* in F-II_1_ (M1: NM_000274.4 c.1186C>T, p.R396*; M2: NM_000274.4 c.748C>T, p.R250*) and a heterozygous *OAT* in F-I_2_ (M1: NM_000274.4 c.1186C>T, p.R396*) (Figure 1A,B). Because WES may miss deep intron variants or structural variants which can influence the phenotype, WGS was performed on F-I_2_. WGS did not identify any other *OAT* pathogenic mutations, nor any pathogenic mutation in other genes that may be associated with her clinical phenotype. The pathogenicity analysis of the identified variants is shown in Table S2. The amino acid residues of the identified variants were highly conserved among the species (Figure 1C). The three-dimensional structures of the wild-type and variant OAT proteins are shown in Figure 4A; both variant proteins were predicted to be shorter than the wild-type protein (493 amino acids). The catalytic sites for substrate specificities in the OAT protein are shown in Figure 4B in stick mode, which shows that the nonsense variant of M2 at position 250 was deleterious due to the loss of the catalytic sites due to early termination of the protein translation.

### 3.4. OAT mRNA Detection

Figure 4C shows that the relative *OAT* mRNA expression levels in F-II_1_ and F-I_2_ were approximately only 29% and 46%, respectively, of those in normal individuals. Statistically significant differences were found between F-II_1_ and the normal controls (*n* = 3, *p* < 0.001); F-I_2_ and the normal controls (n = 3, *p* < 0.001); and F-II_1_ and F-I_2_ (n = 3, *p* = 0.003).

Sanger sequencing of *OAT* cDNA on M1 (c.1186C>T) indicated that the remaining transcript in F-I_2_ is mainly due to the WT allele (Figure 4D). The remaining transcript in F-II_1_ from the WT allele is virtually undetectable (Figure 4D). Sanger sequencing of *OAT* cDNA on M2 (c.748C>T) indicated that the remaining transcript in F-II_1_ from the mutated allele is virtually undetectable (Supplementary Table S2).

Furthermore, according to the results of NGS for the transcription products, mutation M2 (c.748C>T) accounts for 12.53% of the transcript in F-II_1_; mutation M1 (c.1186C>T) accounts for 40.4% of the transcript in F-I_2_; and mutation M1 (c.1186C>T) accounts for 82.89% of the transcript in F-II_1_.

### 3.5. Treatment and Follow-Up

An arginine-restricted diet and vitamin B6 supplementation were administered to both individuals. Serum Orn levels of F-II_1_ progressively decreased to 209.68 µM, 194.42 µM, and 132.71 µM during each 3-month follow-up period, while the serum Orn levels of F-I_2_ decreased to 92.60 µM, 80.03 µM, and 74.97 µM. During the follow-up, fundus lesions, including the macular structure of F-II_1_, remained bilaterally stable.

## 4. Discussion

In this study, we identified a Chinese family in which the proband was diagnosed with GACR; two previously reported *OAT* variants (c.1186C>T and c.748C>T) were found. Interestingly, the girl’s mother, who only had one *OAT* variant (c.1186C>T) following the genome sequencing analysis, displayed some ocular manifestations (bilateral pathologic myopia and posterior staphyloma, atrophy of retina and choroidal capillary layer, RPE abnormalities of the right eye) and a slightly elevated serum Orn level, indicating that she might be a carrier with a mild phenotype caused by the presence of the heterozygous mutation. Additionally, the relative mRNA expression levels of *OAT* were decreased in both the mother and daughter. Accordingly, this report is the first to report a carrier presenting with a mild phenotype of GACR.

GACR is a rare progressive autosomal recessive disorder. With an error in the Orn metabolism attributed to OAT deficiency, ocular manifestations mainly manifest as the aggravation of gyrate chorioretinal atrophy with age, which significantly threatens vision and frequently leads to blindness. In this study, the proband (F-II_1_) manifested typical multiple sharply demarcated, scallop-shaped chorioretinal atrophy, high myopia, and bilateral posterior staphyloma. In particular, hyperreflective deposits below the RPE layer occurred in areas where the retina and choroid had not yet atrophied, consistent with previous theories showing that Orn cytotoxicity in ornithine-δ-aminotransferase-deficient human RPE cells could be the main pathogenic mechanism of GACR in an in vitro model [25,26]. The participant’s accumulation of serum Orn levels was higher than the reference value. Previous studies have demonstrated the effectiveness of an arginine-restricted diet and vitamin B6 supplementation for GACR [20]. Similarly, after the definitive diagnosis and initiation of treatment, the serum Orn levels began to decrease, and the macular abnormalities were stable. Notably, the daughter had a history of long-term follow-up at an optometry clinic. However, her fundus manifestations were ignored for a long time until macular abnormalities appeared. A thorough examination and follow-up of the fundus, including the peripheral fundus, is suggested for children with uncommon rapid myopia progression and poor BCVA. Fundus diseases include not only GACR but also familial exudative vitreoretinopathy [27], vitreoretinal degeneration diseases (such as Stickler Syndrome [28] and Wagner syndrome [29]), and some other inherited choroidal retinal diseases, which should be considered in these children. The diagnosis of GACR should be confirmed not only by thorough multimodal fundus imaging but also by the presence of hyperornithinaemia and *OAT* variants. Accurate identification of genetic causes allows for timely diagnosis and early treatment to protect the vision of patients with GACR.

The mother was a heterozygous carrier with only one variant allele of *OAT*, as confirmed by WGS. As the patient complained of ocular discomfort, detailed ophthalmological examinations were performed, which revealed unilateral severe myopia and bilateral posterior staphyloma, as well as atrophy of the retina and the choroidal capillary layer and PED with an area of disorganized RPE layer in her right eye, which led us to suspect a mild phenotype of GACR. Subsequent serum metabolic tests also showed the mild elevation of Orn. As mentioned, RPE was the most vulnerable in GACR; moreover, we also detected PED and an area of disorganized RPE layer adjacent to the boundary of one cilioretinal atrophy lesion. Further, the patient’s right eye showed high myopia, which is a common manifestation of GACR. Her left eye had an axial length of only 24.52 mm but also showed posterior staphyloma. Previous studies have reported that damaged RPE cells may be associated with posterior staphyloma formation. On one hand, damaged RPE cells would allow the passive spreading of the remaining cells, stretching the retina in the posterior pole and compressing the choroid between the expanding Bruch’s membrane and the sclera [30,31]; on the other hand, the reduction of oxygen demand by RPE cells and photoreceptors might lead to vascular constriction and thinning at the level of the choroid, stimulating posterior staphyloma development [32]. These might be the reasons for the phenotypes found in the proband’s mother.

This phenomenon of “affected” carriers in autosomal recessive conditions is not novel, and they have mainly been detected in inherited metabolic abnormalities diseases. For instance, carriers for phenylketonuria only have 13–42% of enzymatic activity as compared with the normal controls who do not carry any pathogenic variants of *PAH* genes (100% enzymatic activity) in liver biopsy studies [14,15,33,34]. Furthermore, for cystic fibrosis, some studies indicate that carriers are at increased risk for some conditions associated with cystic fibrosis, including pancreatitis, male infertility, bronchiectasis, and cholelithiasis [16,35]. Contrary to the carriers who are frequently described as “unaffected,” the concept of allele-dose responses indicate that they might actually present with an abnormal biochemical phenotype, which may lead to mild clinical phenotypes [36]. However, the proportion of carriers who develop mild phenotypes still remains very low. Meanwhile, differing from general theories of allele-dose responses effects triggered by heterozygous variants, other possible mechanisms for the manifestations of heterozygotes in recessive conditions also exist. For example, in PKU, the likelihood is that a mutant copy of the protein may be having an effectively dominant effect where this combines with wild-type copies in a multimeric protein [33,34]. Thus, suspected carriers of mild phenotypes require more intensive and comprehensive testing and more considered judgement. There are no prior reports of carriers presenting with a clinical phenotype of GACR. We hypothesize that this might be due to the low proportion of carriers presenting with phenotypes and the lack of serious complaints of vision loss; thus, these carriers may neglect undergoing detailed ophthalmologic examinations. Given that the mother in this study and other similar carriers could reduce their risks of serious complications and prevent damage from genetic disease, we hope that there will be more research studies to establish the prevalence of carriers with phenotypes in recessive genetic disorders where there might be treatment benefits. Meanwhile, if conditions permit, we recommend that individuals who are heterozygous for recessive genetic disorders for which there are published reports of carriers with phenotypes should be evaluated and screened in the future to determine possible clinical implications.

To further investigate the association between the mother’s phenotype and the *OAT* variant, the transcription of *OAT* gene in both the mother and daughter were analysed. Compared with that in the normal control, the relative *OAT* mRNA expression levels of both mother and daughter were decreased. The remaining transcript of F-II_1_ in the variant M1 (c.1186C>T) is mainly due to the wild-type allele as 59.6%, while the remaining transcript of F-I_2_ in the variant M2 (c.748C>T) is also mainly due to the wild-type allele as 87.47%, indicating that the transcription of the *OAT* gene is impaired and both nonsense mutations of M1 and M2 may lead to mRNA degradation via nonsense-mediated decay (NMD). NMD is a transcriptional regulatory mechanism that detects mRNA with premature termination codons and triggers truncated protein degradation to prevent potentially harmful effects [37]. Thus, *OAT* mRNA degradation via NMD may be a pathogenic cause of GACR. Moreover, mutated cDNA in M1 of F-II_1_ accounts for 82.89% of the transcription products, indicating that M2 (p.R250*) may cause more severe NMD than M1 (p.R396*). The differences between the mother and daughter may suggest an association with phenotypic severity. Given the difficulty of detecting ornithine-δ-aminotransferase activity within target tissues, including the eyes, muscles, and nerves in patients with GACR, the relative *OAT* mRNA expression of peripheral blood may not only identify the pathogenicity but also indicate the processes and prognosis, although this will require further study and multivariate analyses.

There are some limitations to the present study. Due to the rarity of the disease, we did not observe enough cases of carriers with GACR, and it would be preferable to measure OAT enzyme activity. Additionally, information on this family was still partly missing because the father was not available for examination.

## 5. Conclusions

In conclusion, to the best of our knowledge, this study reports for the first time a carrier with one variant allele of *OAT* who exhibited mild GACR phenotypes; this suggests that family members should be aware of the possible involvement of heterozygous variants in autosomal recessive conditions. Furthermore, the triggering of *OAT* mRNA degradation by NMD may be the causative mechanism of GACR. The present study deepens understanding of this disease, while emphasizing the importance of fundus screening in children with high myopia.

## Figures and Tables

**Table 1:** Ocular manifestation and the results of the haematological test.

		F-II_1_		F-I_2_	
Age (Years)		19		54	
Chief complaint		Decreased vision bilaterally		High myopia and fluttering dark shadows in the right eye	
Ophthalmic parameters		OD	OS	OD	OS
	IOP (mmHg)	12.6	12.4	14.5	13.6
	AL (mm)	29.40	29.40	26.45	24.52
	SE→BCVA	−17.00 D→20/66	−18.25 D→20/66	−9.00 D→20/28	−2.75 D→20/28
	Ocular manifestations	Punctate posterior subcapsular cataract	/	Nuclear cataract	Nuclear cataract
		Bilateral high myopia Bilateral PS (Type 1) Bilateral sharply demarcated circular areas of peripheral chorioretinal atrophy Bilateral macular atrophy		High myopia PS (Type 1) Focal circular areas of retinal atrophy /	Mild myopia Inferior PS (Type 5) / /
Haematological testing (μM)		Serum value		Serum value	Reference
	Ornithine	257.92 ↑		102.08 ↑	10–100
	Creatine	94.83 ↓		121.75	95–1000

Abbreviations: IOP: intraocular pressure; AL: Axial length; SE: spherical equivalent, = sphere + cylinder/2 D; BCVA: best corrected visual acuity; PS: posterior staphyloma; OD: the right eye; OS: the left eye. Type 1: Wide, macular PS; Type 5: Inferior PS. “↑” indicates an elevated value, while “↓” indicates a decreased value.

## Data Availability

Data are available from the authors upon reasonable request.

## Supplementary Materials

The following supporting information can be downloaded at https://www.mdpi.com/article/10.3390/genes15081020/s1: Figure S1: Anterior segment photographs of F-II1 (right eye); Table S1: Sequences of qRT-PCR primers. Table S2. Pathogenicity prediction of the identified mutations.

## References

[1] Tsang S.H., Aycinena A.R.P., Sharma T. (2018). Inborn errors of metabolism: Gyrate atrophy. Adv. Exp. Med. Biol..

[2] Montioli R., Bellezza I., Desbats M.A., Borri Voltattorni C., Salviati L., Cellini B. (2021). Deficit of human ornithine aminotransferase in gyrate atrophy: Molecular, cellular, and clinical aspects. Biochim. Biophys. Acta. Proteins Proteom..

[3] Ramesh V., Benoit L.A., Crawford P., Harvey P.T., Shows T.B., Shih V.E., Gusella J.F. (1988). The ornithine aminotransferase (OAT) locus: Analysis of RFLPs in gyrate atrophy. Am. J. Hum. Genet..

[4] Kaiser-Kupfer M.I., Valle D., Del Valle L.A. (1978). A specific enzyme defect in gyrate atrophy. Am. J. Ophthalmol..

[5] Hayasaka S., Kodama T., Ohira A. (2011). Retinal risks of high-dose ornithine supplements: A review. Br. J. Nutr..

[6] Peltola K.E., Näntö-Salonen K., Heinonen O.J., Jääskeläinen S., Heinänen K., Simell O., Nikoskelainen E. (2001). Ophthalmologic heterogeneity in subjects with gyrate atrophy of choroid and retina harboring the L402P mutation of ornithine aminotransferase. Ophthalmology.

[7] Katagiri S., Gekka T., Hayashi T., Ida H., Ohashi T., Eto Y., Tsuneoka H. (2014). OAT mutations and clinical features in two Japanese brothers with gyrate atrophy of the choroid and retina. Doc. Ophthalmol..

[8] Mansour A.M., Elnahry A.G., Tripathy K., Foster R.E., Mehanna C.J., Vishal R., Çavdarlı C., Arrigo A., Parodi M.B. (2021). Analysis of optical coherence angiography in cystoid macular oedema associated with gyrate atrophy. Eye.

[9] Tripathy K., Chawla R., Sharma Y.R., Gogia V. (2016). Ultrawide field fluorescein angiogram in a family with gyrate atrophy and foveoschisis. Oman J. Ophthalmol..

[10] Kaiser-Kupfer M.I., de Monasterio F., Valle D., Walser M., Brusilow S. (1981). Visual results of a long-term trial of a low-arginine diet in gyrate atrophy of choroid and retina. Ophthalmology.

[11] McInnes R.R., Arshinoff S.A., Bell L., McCulloch C. (1980). Treatment of gyrate atrophy of the choroid and retina with low arginine diet. Trans. Am. Ophthalmol. Soc..

[12] Bergen A.A., Buijs M.J., Ten Asbroek A.L., Balfoort B.M., Boon C.J., Brands M.M., Wanders R.J., van Karnebeek C.D., Houtkooper R.H., Dutch GACR “Bird’s Eye View” Consortium (2024). Vision on gyrate atrophy: Why treat the eye?. EMBO Mol. Med..

[13] Hames A., Khan S., Gilliland C., Goldman L., Lo H.W., Magda K., Keathley J. (2023). Carriers of autosomal recessive conditions: Are they really “unaffected?”. J. Med. Genet..

[14] Hillert A., Anikster Y., Belanger-Quintana A., Burlina A., Burton B.K., Carducci C., Chiesa A.E., Christodoulou J., Đorđević M., Desviat L.R. (2020). The genetic landscape and epidemiology of phenylketonuria. Am. J. Hum. Genet..

[15] van Spronsen F.J., Blau N., Harding C., Burlina A., Longo N., Bosch A.M. (2021). Phenylketonuria. Nat. Rev. Dis. Primers.

[16] Polgreen P.M., Comellas A.P. (2022). Clinical phenotypes of cystic fibrosis carriers. Annu. Rev. Med..

[17] Wagenaar M., ter Rahe B., van Aarem A., Huygen P., Admiraal R., Bleeker-Wagemakers E., Pinckers A., Kimberling W., Cremers C. (1995). Clinical findings in obligate carriers of type I Usher syndrome. Am. J. Med. Genet..

[18] Ohno-Matsui K. (2014). Proposed classification of posterior staphylomas based on analyses of eye shape by three-dimensional magnetic resonance imaging and wide-field fundus imaging. Ophthalmology.

[19] Curtin B.J. (1977). The posterior staphyloma of pathologic myopia. Trans. Am. Ophthalmol. Soc..

[20] Ohno-Matsui K., Wu P.C., Yamashiro K., Vutipongsatorn K., Fang Y., Cheung C.M.G., Lai T.Y.Y., Ikuno Y., Cohen S.Y., Gaudric A. (2021). IMI Pathologic Myopia. Investig. Ophthalmol. Vis. Sci..

[21] Ju Y., Zhang L., Gao F., Zong Y., Chen T., Ruan L., Chang Q., Zhang T., Huang X. (2024). Genetic characteristics and clinical manifestations of foveal hypoplasia in familial exudative vitreoretinopathy. Am. J. Ophthalmol..

[22] Lei C., Liao K., Zhao Y., Long Z., Zhu S., Wu J., Xiao M., Zhou J., Zhang S., Li L. (2023). A novel system for the detection of spontaneous abortion-causing aneuploidy and its erroneous chromosome origins through the combination of low-pass copy number variation sequencing and NGS-based STR tests. J. Clin. Med..

[23] Richards S., Aziz N., Bale S., Bick D., Das S., Gastier-Foster J., Grody W.W., Hegde M., Lyon E., Spector E. (2015). Standards and guidelines for the interpretation of sequence variants: A joint consensus recommendation of the American College of Medical Genetics and Genomics and the Association for Molecular Pathology. Genet. Med..

[24] Livak K.J., Schmittgen T.D. (2001). Analysis of relative gene expression data using real-time quantitative PCR and the 2-ΔΔCT Method. Methods.

[25] Ohnaka M., Okuda-Ashitaka E., Kaneko S., Ando A., Maeda M., Furuta K., Suzuki M., Takahashi K., Ito S. (2011). Induction of arginase II mRNA by nitric oxide using an in vitro model of gyrate atrophy of choroid and retina. Investig. Ophthalmol. Vis. Sci..

[26] Sergouniotis P.I., Davidson A.E., Lenassi E., Devery S.R., Moore A.T., Webster A.R. (2012). Retinal structure, function, and molecular pathologic features in gyrate atrophy. Ophthalmology.

[27] Tauqeer Z., Yonekawa Y. (2018). Familial exudative vitreoretinopathy: Pathophysiology, diagnosis, and management. Asia-Pac. J. Ophthalmol..

[28] Snead M.P., Yates J.R. (1999). Clinical and molecular genetics of Stickler syndrome. J. Med. Genet..

[29] Araújo J.R., Tavares-Ferreira J., Estrela-Silva S., Rocha P., Brandão E., Faria P.A., Falcão-Reis F., Rocha-Sousa A. (2018). Wagner syndrome: Anatomic, functional and genetic characterization of a Portuguese family. Graefes Arch. Clin. Exp. Ophthalmol..

[30] Ohno-Matsui K., Jonas J.B. (2019). Posterior staphyloma in pathologic myopia. Prog. Retin. Eye Res..

[31] Komori S., Ueno S., Ito Y., Sayo A., Meinert M., Kominami T., Inooka D., Kitagawa M., Nishida K., Takahashi K. (2019). Steeper macular curvature in eyes with non-highly myopic retinitis pigmentosa. Investig. Ophthalmol. Vis. Sci..

[32] El Matri L., Falfoul Y., El Matri K., El Euch I., Ghali H., Habibi I., Hassairi A., Chaker N., Schorderet D., Chebil A. (2020). Posterior staphylomas in non-highly myopic eyes with retinitis pigmentosa. Int. Ophthalmol..

[33] Kaufman S., Max E.E., Kang E.S. (1975). Phenylalanine hydroxylase activity in liver biopsies from hyperphenylalaninemia heterozygotes: Deviation from proportionality with gene dosage. Pediatr. Res..

[34] Berry H.K., Hsieh M.H., Bofinger M.K., Schubert W.K. (1982). Diagnosis of phenylalanine hydroxylase deficiency (phenylketonuria). Am. J. Dis. Child..

[35] Miller A.C., Comellas A.P., Hornick D.B., Stoltz D.A., Cavanaugh J.E., Gerke A.K., Welsh M.J., Zabner J., Polgreen P.M. (2020). Cystic fibrosis carriers are at increased risk for a wide range of cystic fibrosis-related conditions. Proc. Natl. Acad. Sci. USA.

[36] Keathley J., Garneau V., Zavala-Mora D., Heister R.R., Gauthier E., Morin-Bernier J., Green R., Vohl M.C. (2021). A systematic review and recommendations around frameworks for evaluating scientific validity in nutritional genomics. Front. Nutr..

[37] Karousis E.D., Mühlemann O. (2019). Nonsense-mediated mRNA decay begins where translation ends. Cold Spring Harb. Perspect. Biol..

