# Supplementary material for: Mild Phenotypes of Gyrate Atrophy in a Heterozygous Carrier with One Variant Allele of OAT
Source: Genes (Basel). 2024 Aug 2;15(8):1020. doi: 10.3390/genes15081020 (PMC11353755; doi:10.3390/genes15081020)
Supplement: Supplementary file 1 [file genes-15-01020-s001.zip › Supplementary_Materials.pdf]

Supplementary Materials

Supplementary Figure S1. Anterior segment photographs of F-II1 (right eye). Following mydriasis, the anterior segment photograph of the right eye was captured using a photographic system (Keeler, Windsor, UK). Red arrows corresponded to punctate posterior capsular opacification.

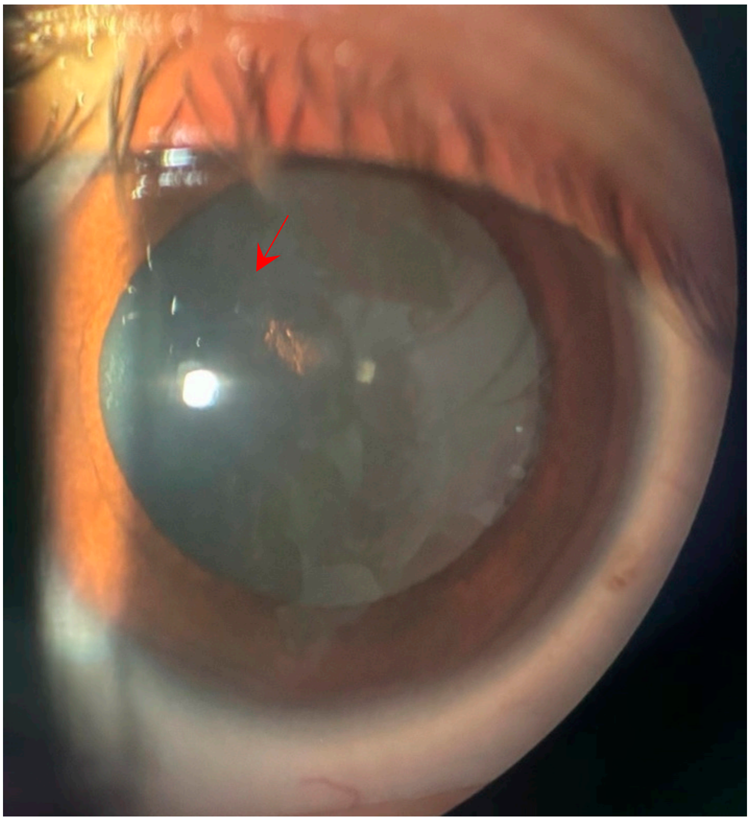

Supplementary Table S1. Sequences of qRT-PCR primers.

Gene	Primer	Primer sequence (5'-3')
OAT	KP1011-F	ACTGCCGTAAGAGGAAAAGGA
	KP1011-R	ATCCTCCTTGATCACCAGCG
GAPDH	KP0713-F	AAATCAAGTGGGGCGATGCT
	KP0713-R	GATGACCCTTTTGGCTCCCC

Abbreviations: qRT-PCR: quantitative real-time polymerase chain reaction

Supplementary Table S2. Pathogenicity prediction of the identified mutations

Content	*OAT*: NM_000274.4 c.1186C>T, p.R396*;		*OAT*: NM_000274.4 c.748C>T, p.R250*;	
	**Prediction**	**Score**	**Prediction**	**Score**
**LRT**	Deleterious	0	Deleterious	0
**FATHMM-MKL**	Deleterious	0.967	Deleterious	0.767
**ClinPred**	Deleterious	0.966	Deleterious	0.997
**CADD**	Deleterious	8.681	Deleterious	7.518
**DANN**	Deleterious	0.997	Deleterious	0.998
**GERP**	Conserved	5.07	Conserved	2.76
**Mutation Taster**	Disease causing automatic	1	Disease causing automatic	1
**SPIDEX**	Disruption	-31.0975	Disruption	-68.9126
**Reference**	Reported		Reported	
**ACMG**	Likely pathogenic		Pathogenic	

Supplementary Annex S1. Sanger Sequencing and Next-generation Sequencing raw data against transcription products of two family members and normal controls
